# Successful transureteropyelostomy after heminephrectomy of a bilateral hydronephrotic horseshoe kidney: a case report

**DOI:** 10.1186/1752-1947-2-231

**Published:** 2008-07-16

**Authors:** Holger Gerullis, Christoph Eimer, Dietmar Betz, Thomas Otto

**Affiliations:** 1 Urology Department, Lukas Hospital, Preussenstrasse, Neuss, 41464, Germany

## Abstract

**Introduction:**

Horseshoe kidney is a rare congenital malformation that is found in approximately 0.25% of the general population and usually remains asymptomatic.

**Case presentation:**

We report a successful transureteropyelostomy after heminephrectomy of the non-functional right moiety in a 25-year-old man with horseshoe kidney who had a combined 50% functional loss and hydronephrosis due to multiple distal ureteral strictures on the functionally remaining left side. Continuous ureteral stenting of the remaining part of the former horseshoe kidney was avoided during a follow-up of 2 years.

**Conclusion:**

Urologists are often faced with technically difficult cases that are not responsive to standard operative procedures, and this case illustrates an individual surgical approach in a clinical situation.

## Introduction

A horseshoe kidney is a rare non-fatal congenital malformation of renal development. It usually remains asymptomatic and in many cases it is discovered incidentally. This anomaly is found twice as often in men as in women. It may predispose the patient to various symptoms and functional disorders such as abdominolumbar pain, renal stones, ureteropelvic junction obstruction and hydronephrosis or pyonephrosis [1]. A thorough urologic evaluation should be initiated in diagnosed cases. Here we report a case of successful surgical treatment of a horseshoe kidney with combined 50% functional loss and hydronephrosis on the functionally remaining side.

## Case presentation

A 25-year-old patient presented to our institution with a horseshoe kidney. Diagnostic work-up was initiated for persistent abdominolumbar pain after surgical reduction of an umbilical hernia 1 year prior to presentation. The patient presented with a further history of a partial left hydronephrosis, for which he had been treated with a double J (DJ) ureteral stent. At that time no further functional diagnostic studies were performed and the patient was lost to follow-up for 1 year.

**Figure 1:**
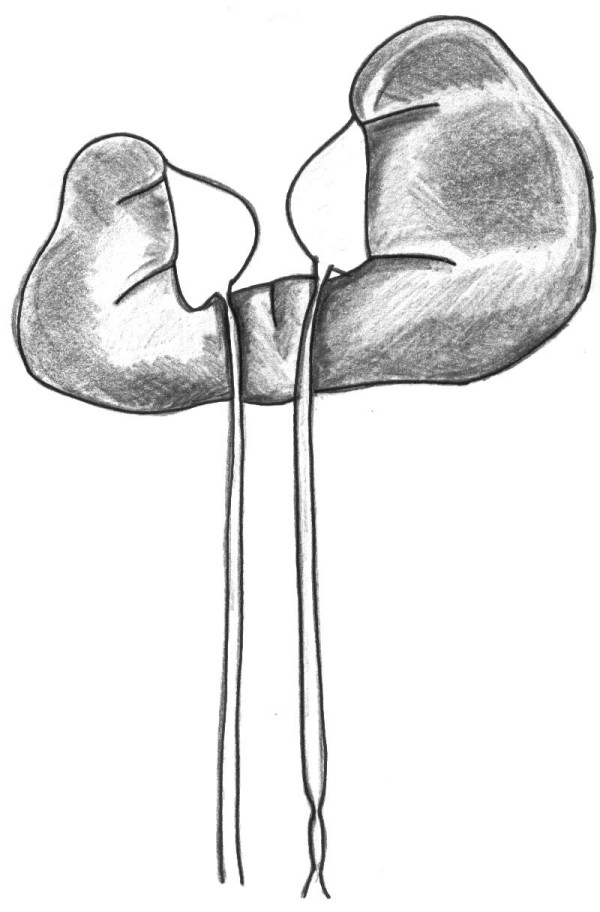
**Preoperative situation**. The atrophic right moiety of the horseshoe kidney with uretero-pelvic junction obstruction, consecutive hydronephrosis and normal distal right ureter are shown. The left moiety showing hydronephrosis due to a relative stricture of uretero-pelvic junction in combination with two distal ureteral strictures can also be seen (*in situ *ureteral stent not shown).

On presentation, ultrasound examination revealed a hydronephrosis grade III of the right and grade I to II of the left moiety of the horseshoe kidney. A DJ stent was noted in an orthotopic position on the left side. On examination the patient showed mild right flank pain. Haematological examination was unremarkable except for a slightly increased serum creatinine of 1.23 mg/dl. Retrograde ureteropyelography of the right moiety revealed hydronephrosis with abrogated calix structures. The left ureter showed multiple distal strictures and a stenosis of the left ureteropelvic junction was suspected (Figure 1). Vesico-ureteral reflux could be excluded radiologically. There was no evidence of renal stones on X-ray or intra-operatively.

Tc^99 m ^mercaptoacetyltriglycine scintigraphic examination detected a functional repartition of 0% (right) and 100% (left); the total tubular extraction rate was reduced to 207 ml/min per 1.73 qm.

**Figure 2:**
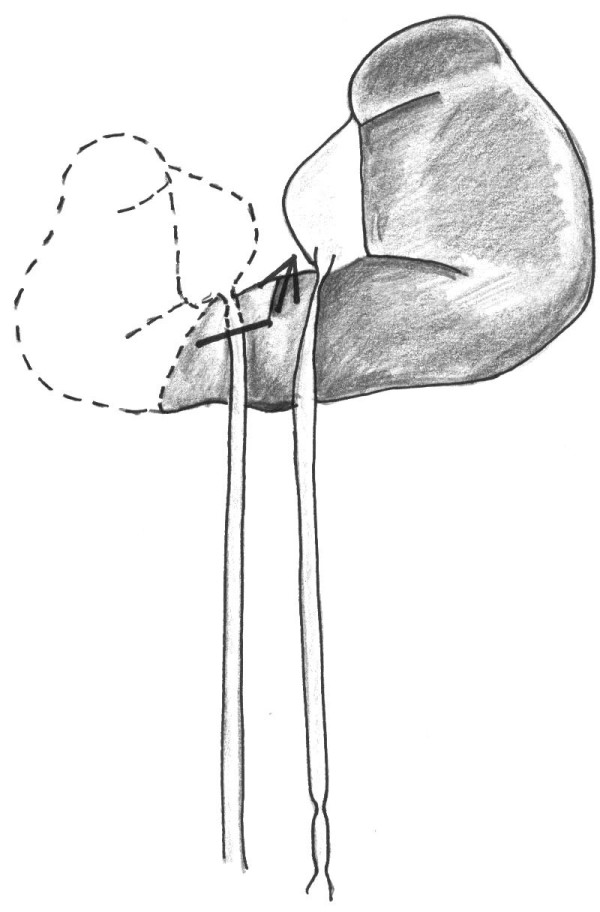
**Intraoperative situation. ** Heminephrectomy of the right moiety with dissection of the right ureter, excision of the uretero-pelvic stricture followed by transposition of the right ureter to the left pelvis (transureteropyelostomy).

**Figure 3:**
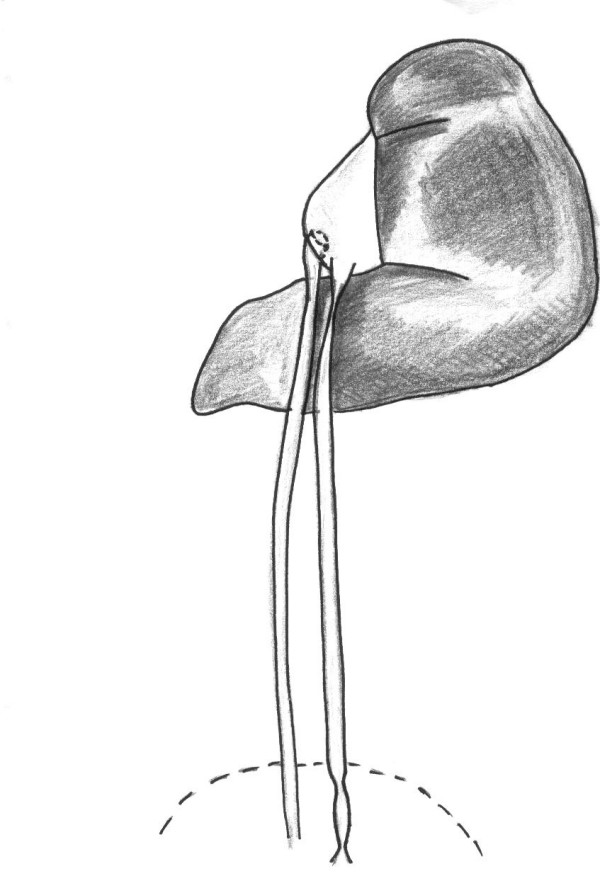
**Postoperative situation with improvement of urine drainage of the left pelvis via two ureters**. Residual hydronephrosis was regressive after 2 months.

We performed a nephrectomy of the right hydronephrotic part of the kidney using open surgery (Figure 2). Histological examination revealed chronic interstitial nephritis without evidence of neoplasia. In order to conserve the left part of the kidney, and to guarantee optimal drainage of the pelvis using the remaining right ureter, a transureteropyelostomy was successfully undertaken. Both ureters were splinted using DJ stents. No severe complications were observed during the entire postoperative period. The ureteral stents were removed 2 months after the initial operative procedure (Figure 3). Postoperative intravenous pyelography revealed no hydronephrosis of the double-drained pelvis. During a follow-up period of over 1 year there was no need for continuous ureteral stenting of the remaining part of the former horseshoe kidney.

## Discussion

Horseshoe kidney can be found with an incidence of 1 in 304 in the general population, with an increased incidence in men [1]. Embryologically it represents the most common failure of migration and rotation of the metanephric buds from their pelvic position during the fourth to sixth week of gestation. The first description of a horseshoe kidney as a pathology of the kidney came from Morgagni in 1820. Hydronephrosis is a common complication of horseshoe kidney [2]. While most horseshoe kidneys remain asymptomatic, hydronephrosis can cause irreversible functional damage to the organ. Surgical treatment of the functionally reduced or even non-functional part of the kidney can therefore become inevitable [3].

In the reported case, the initial indication for surgery was the resection of the non-functioning right moiety of the horseshoe kidney in order to prevent chronic infections and avoid recurrent flank pain. The intention of preservation and functional supply of the remaining right ureter led to transureteropyelostomy. This surgical approach has been described and reported in only a few cases [4]. Ponthieu and colleagues commented on five cases where the indication for this surgery was extensive loss of substance of the distal ureter due to radiation fibrosis, operative trauma or tumour invasion [5]. As the extent of the lesions made vesical anastomosis impossible, the described surgical approach was chosen. Unlike Ponthieu et al. we did not face the problem of transpositioning a short ureter. In addition, the patients described by Ponthieu et al. maintained both kidneys and the transureteropyelostomy was performed in order to fashion a duplicated joining excretion system via one remaining distal ureter. In contrast, our surgical approach was to assure a duplicated draining system with two remaining distal ureters for the remaining moiety of the horseshoe kidney. This individual modification of transureteropyelostomy has not been reported so far.

In cases of ureteric strictures, long-term treatment with *in situ *ureteral stents should be avoided whenever possible; in particular, a definitive surgical procedure should be the aim in young patients where the risks associated with anaesthesia are low. Several treatment options for ureteric strictures, such as transient stenting, repair with intrinsic urinary tract tissues and partial or total ureteral replacement are described [6-8]. As the orthotopic right ureter showed no signs of stricture or vesico-ureteral reflux the described surgical technique was sufficient for drainage of the left pelvis without performing an antirefluxive technique such as ureteroneocystostomy. We believe that the restoration of urothelial continuity using accessible intrinsic, if possible orthotopic, tissue is the best treatment option for ureteric strictures.

Transureteroureterostomy (TUU) was not used in this particular case. The established general criteria for TUU, as of a normal donor kidney and upper ureter on one side and a normal recipient ureter and bladder, are different from the situation in this case [9]. In addition, since TUU may be associated with serious complications, including failure of the uretero-ureteral anastomosis as well as potential injury to both donor and recipient upper tracts, we preferred transureteropyelostomy in order to decrease the risk of postoperative morbidity [10].

The described procedure is a credible example of the subjective and objective superiority of the definitive surgical approach in order to avoid prolonged ureteral stenting and possible complications [11]. A systematic approach and procedure for dealing with complex ureteral problems and congenital malformations should always leave space for individual therapeutic strategies to ensure the highest benefit for the patients in their particular circumstances.

## Conclusion

Horseshoe kidney is a congenital malformation which may predispose the patient to numerous complications including hydronephrosis and loss of renal function. Various complications of long-term ureteral stenting have been reported. Whenever possible, long-term stenting of the ureter should be avoided and a definitive therapeutic approach should be the goal, especially in patients with good general health.

Urologists are often faced with technically difficult cases that are not responsive to standard operative procedures, and this case illustrates an individual surgical approach in a very particular clinical situation. It highlights the improvement of the patient's quality of life as well as the long-term functional protection of the remaining part of the horseshoe kidney.

## Abbreviations

DJ: Double J; TUU: Transureteroureterostomy.

## Competing interests

The authors declare that they have no competing interests.

## Consent

Written informed consent was obtained from the patient for publication of this case report and accompanying images. A copy of the written consent is available for review by the Editor-in-Chief of this journal.

## Authors' contributions

HG drafted the manuscript, performed a literature review and participated in the surgery. CE participated in the surgery and was involved in the clinical follow-up. DB performed the surgery. TO supervised this report. All authors read and approved the final manuscript.
